# Data-science ready, multisite, human diffusion MRI white-matter-tract statistics

**DOI:** 10.1038/s41597-020-00760-3

**Published:** 2020-11-30

**Authors:** Garikoitz Lerma-Usabiaga, Pratik Mukherjee, Michael L. Perry, Brian A. Wandell

**Affiliations:** 1 Department of Psychology, Stanford University, 450 Jane Stanford Way, Jordan Hall Building, 94305 Stanford, California USA; 2 BCBL. Basque Center on Cognition, Brain and Language, Mikeletegi Pasealekua 69, Donostia - San Sebastián, 20009 Gipuzkoa Spain; 3 Wu Tsai Neurosciences Institute, Stanford University, 94305 Stanford, California USA; 4 Radiology and Biomedical Imaging, University of California, San Francisco, California USA; 5 Bioengineering and Therapeutic Sciences, University of California, San Francisco, California USA

**Keywords:** Cognitive neuroscience, Brain, Biophysical models, Brain imaging, Magnetic resonance imaging

## Abstract

The white matter tracts in the living human brain are critical for healthy function, and the diffusion MRI measured in these tracts is correlated with diverse behavioral measures. The technical skills required to analyze diffusion MRI data are complex: data acquisition requires MRI sequence development and acquisition expertise, analyzing raw-data into meaningful summary statistics requires computational neuroimaging and neuroanatomy expertise. The human white matter study field will advance faster if the tract summaries are available in plain data-science-ready format for non-diffusion MRI experts, such as statisticians, computer graphic researchers or data scientists in general. Here, we share a curated and processed dataset from three different MRI centers in a format that is data-science ready. The multisite data we share include measures of within and between MRI center variation in white-matter-tract diffusion measurements. Along with the dataset description and summary statistics, we describe the state-of-the-art computational system that guarantees reproducibility and provenance from the original scanner output.

## Abstract (summary)

**Table:** 

Measurement(s)	white matter • functional brain measurement • brain measurement • brain connectivity measurement
Technology Type(s)	Diffusion Weighted Imaging • magnetic resonance imaging (MRI) • computational modeling technique
Factor Type(s)	fractional anisotropy (fa) • axial diffusivity (ad) • mean diffusivity (md) • volume • radial diffusivity (rd) • torsion • curvature
Sample Characteristic - Organism	Homo sapiens

Machine-accessible metadata file describing the reported data: 10.6084/m9.figshare.13076033

## Background & Summary

We describe and share the diffusion MRI white-matter tract modelling and summary statistics that were used to evaluate how well a diffusion scientific observation translates into a clinical setting^1^. In our publication we used only a fraction of the information and performed a fraction of the potential analyses with the dataset shared here. The data are derived from diffusion-weighted MRI measurements in 187 human subjects from three different MRI Centers with instruments from two different vendors and several pulse sequences. The data we share are offered in a format that is readily accessible to data scientists who would like to explore the properties of white-matter-tract estimates.

**Fig. 1:**
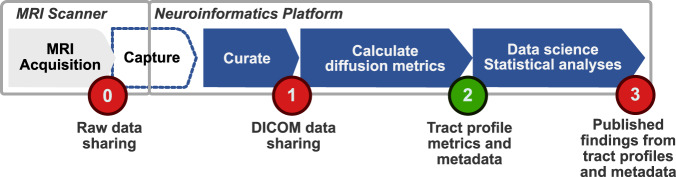
The analysis stages from human diffusion-weighted imaging tractography pipeline. The image shows a series of analysis steps from the MRI diffusion-weighted acquisition to the final publication of figures and tables. (0) The raw data acquired from the MRI scanner are converted into DICOM images by custom software provided by the instrument manufacturer. (1) These DICOMS files include important metadata about the instrumental parameters. (2) The DICOM files are converted into diffusion orientation distribution functions, and these are then analyzed for white-matter streamlines. The subject metadata and parameters of the algorithms are added as additional metadata. This dataset is shared at this stage. (3) The diffusion data and tracts are further analyzed using data science methods to achieve new scientific insights. These results are usually shared in publications as Figures and Tables. Qualified investigators who wish to examine the DICOM data (1) can contact the authors. This publication shares to all research investigators the data and metadata at stage (2). The containers with the software that performed the calculations from DICOM to streamlines are public.

The raw data from an MR scanner comprises sampled measurements in k-space, a Fourier representation (Fig. 1). These raw data are converted by the instrument manufacturers into images that are stored as DICOM files along with critical metadata. The diffusion data are then processed to estimate the diffusion orientation distribution function at each voxel. The voxel data are then used to estimate the presence of major white matter tracts. The MR diffusion metrics along these white-matter tracts (tract profiles) assess how the axons and other cells comprising the tracts restrict diffusion. It is possible to share data at any of these processing steps, and each has value for different purposes. It has been our experience that the numerical calculations of the metrics are of value to many in the data science community. Our collaborators prefer to start with these metrics because the prior steps (conversion to DICOM, preprocessing, voxel-level diffusion modeling, tractography, metric obtention) are quite complex and not their primary target of investigation.

Several research groups requested the tract profile diffusion metrics, which is why we are sharing the dataset in this publication. The profiles of the researchers making this request vary: a big-data-science expert that wanted to find links between white matter and behavioral indexes, a computer vision/image processing/medical imaging expert that wanted to study the individual differences in the geometries of the white matter tracts, a MRI physicist interested in the reproducibility and generalizability of diffusion measurements, or a statistician that wanted to migrate a method developed in genetics to neuroimaging and required a high volume of domain specific data.

The dataset might be of interest for other analyses too:

1. The data includes measures from multiple sites, and these can be difficult to obtain. This opens up new questions that are particularly important for clinicians, although we warn our target researchers that this dataset should not be used for clinical decision-making.
2. The datasets compiled in this dataset were analyzed to understand the generalization of diffusivity measures obtained from different measurement sites^1^. There are many additional analyses that we hope will be explored. These include (a) the reliability of the super-fiber positions and shapes across subjects, (b) regularities and dimensionality of the tract profiles, and (c) analyses of the correlations between tract profiles. Many other analyses are possible, as we explained to the researchers that approached us.
3. The datasets have been carefully curated so that the populations can be compared among them. Same number of subjects, with similar population characteristics. If each one of the datasets is characteristic of the general population, we should obtain the same results in the separate datasets.

Sharing this level of the data makes sense for another reason. Reanalyzing the dataset from the raw or DICOM files repeats a large computation on specialized resources that are not widely available. For example, in this example the DICOM (100 GB), and the intermediate data are approximately 2 TB. The shared dataset is approximately 55 MB, several orders of magnitude less. The analyses that start from the tract profile diffusion metrics are also significant, and these can be performed by a much wider group of research scientists. For those research scientists, we emphasize that the metrics we share have a traceable provenance: the DICOM data are available to any qualified investigator and the computational methods are open-source, containerized and thus reproducible.

The data comprise 231 MRI sessions from 187 subjects. The data were analyzed using the Reproducible Tract Profiles (RTP) tool, which comprises a set of methods to manage and analyze diffusion weighted imaging (DWI) data. The white-matter-tract estimates were computed over 25,000 CPU hours. The RTP tools read MRI data from the scanner and process them through a series of analyses implemented as containers. The methods we used can be re-executed from the DICOM to the tract profiles using the same computations and parameters. Newly acquired data run with the same pipeline can be combined to extend the data shared here. We have successfully run the RTP containers in Docker and Singularity systems, in Mac and Linux, from personal computers to high capacity server clusters.

## Methods

### Datasets

We provide data from 231 MRI sessions, 187 unique subjects, from three different projects:

- **Project WHL:** 44 subjects measured by^2^. We wrote independent computational analyses to identify the tracts and measure diffusion summary statistics. This data comprises one homogeneous unit of 44 subjects.
- **Project HCP:** 44 subjects whose test-retest data are available from the 1200 Human Connectome Project (HCP) release^3^. In both the test and retest sessions b-values 1000, 2000 and 3000 were acquired. This data comprises 6 homogeneous datasets.
- **Project YWM:** 99 subjects measured by^4^. In the same session, two different b-values were acquired. Filtering by age, a subset of 44 subjects can be created to match the other datasets. The data records here include all 99 subjects. This data comprises two homogeneous datasets.

All data provided was obtained following the IRB guidelines of the relevant institutions. See the original publications for details.

Twenty white matter tracts were identified using the Reproducible Tract Profiles algorithm (see below). Eight diffusion imaging metrics were calculated for each tract; these are shared in the data records. The data records also include the metadata about the subject, the scanner / sequence and the analysis configurations. See the data descriptor for more information on the shared dataset.

### Reproducible tract profiles

The complexity of neuroimaging data and analyses challenges researchers’ ability to implement reproducible research methods. In many neuroimaging publications, there is no realistic chance that a reader can replicate the experimental data acquisition or reproduce the computational analyses^5–7^. It is understandable that the costs of replicating an experiment, particularly with a special patient cohort, may be impractical. Even so, it should be possible to share the data and enable colleagues to repeat, check, and explore the computational analysis^8,9^.

The large computational power available to many labs has supported a substantial increase in algorithm complexity. Some of this complexity takes the form of a large number of pipeline parameters that users can set. Choices made in these parameters can have substantial effects on the reported results. In the case of functional MRI (fMRI), the position of the peak activation may range over a cortical area of 25 cm^2^ ^10^. Further^11^, observe that we are often uncertain about critical parameters that must be in computational models. We confirm that this observation also applies to DWI and tractography. It is our experience, too, that scientists find it very difficult to keep track of the full range of parameters used in any particular analysis, and even fewer scientists record the combinations of parameters they used during data exploration^12^.

To address this problem, we created tools that improve computational reproducibility^1^. These methods implement fully reproducible analyses that begin with data acquisition and end with the figures used in the publication (steps 1, 2 and 3 in Fig. 1). The reproducible tract profiles (RTP) methods describe all the computational steps required for a complete tractography solution. This solution is reproducible because the analysis software and its dependencies are encapsulated in a container, and the input data, complete set of analysis configuration parameters and the outputs are stored in a single informatics platform which is installed on a cloud provider. The user accesses the data and controls the computations from the client side using a programming language interface that interacts with the informatics platform through a REST API with the platform. Together with the RTP tools, these methods comprise a system that enables users to check and reproduce all the computations from DWI acquisition to publication.

The methods we used guarantee that each tract profile can be traced to the original DICOMs. The files not shared in this publication (DICOMs and intermediate steps in the calculations) are available for any IRB authorized researcher.

### Computational reproducibility and data provenance

We have maintained the provenance of the dataset and we used reproducible computational methods. For provenance, the raw data from the scanner are immediately placed in a database system. We implement reproducible computational methods by implementing our algorithms as containers. The details of the system we used are as follow:

1. **Capture**: the YWM data were acquired and uploaded directly to the platform with no human interaction. Several tasks are automated in this step, such as DICOM to Nifti conversion, categorization according to acquisition types, and parsing of the data to include metadata within the database. WHL was uploaded from DICOM files, and HCP was uploaded from preprocessed Niftis.
2. **Curate**: once uploaded, the data are available for visualization, computation, sharing, and download. Data can be reorganized, shared with other project members, renamed; most importantly, additional metadata was added. These actions are supported by the web interface or programming language interface in Python and Matlab (the Matlab version we developed is publicly available at https://github.com/vistalab/scitran/). In this stage we make sure that every subject has all the required data and metadata uploaded to the system.
3. **Compute**: The informatics platform provides a system for analyzing the data in a platform-independent, cloud-scale, reproducible manner. Containers and associated JSON files are part of the database and record the computational parameters. Every analysis, including versions, parameters, inputs, and outputs, is named, time-stamped and stored in the searchable database.
4. **Supplementary data**: The DICOM data, metadata and intermediate analysis steps can be made available to anyone with IRB authorization to access the system. For others with IRB authorization but without access to the system the data and derivative files can be exported in BIDS.

#### Container creation and configuration

To guarantee computational reproducibility across platforms we inserted the analysis tools inside of containers (we worked with Docker and Singularity, specifically). The rules for running the container are represented in an associated JSON file that specifies the flags and program parameters (manifest.json). This file contains all the information needed to run the container, such as name, version, labels, description, inputs, outputs, and configuration parameter types and defaults.

To test containers we used the following approach: (1) Run the code directly in Matlab and check the outputs; (2) Compile the Matlab code and run it using Matlab’s Runtime environment (freely downloadable from www.mathworks.com); (3) Build the Docker Container and run it using the default parameters and changed parameters; (4) Check the code into github, tag it, and build the containers in dockerhub; (5) Install the container in our data and computational management system (flywheel.io); (6) Verify that the analysis returns the same results locally and within the data management system.

#### Programmatic access to the data

Increasingly, neuroimaging projects consist of a larger number of subjects. As data scales it becomes increasingly difficult to record and manage configuration parameters and verify software versions that were used to analyze the data. Even with two subjects, it is easy to make mistakes when setting the more than 50 RTP-pipeline config parameters. To manage and verify the computational provenance of the data records, we relied on the ability to programmatically control access to the data and computations in the database.

The code we used is available in https://github.com/vistalab/scitran/. The toolbox provides: *(1) Data/analysis search*, *(2) Metadata management*, *(3) Upload and download, (4) Computational analysis management*, *(5) Information extraction*. Using these methods we can search the data and metadata for specific subjects and analyses. We can then extract the data and metadata used for statistical analyses. The data records shared here were created using these programmatic tools, which guarantees that when using the same version we will generate the same set of tables, as the datatable will be linked to a specific tag in the github code. See the Data Records section for more details.

Researchers can be confident of the reproducibility and provenance of the data we are sharing. They do not need to purchase Matlab or Flywheel, but they will need a computer. The same is true for researcher who want to reproduce our analysis starting with the DICOMs or any intermediate stage in the pipeline.

### Data analysis pipeline

The RTP solution is divided into two main steps: RTP-preproc and RTP-pipeline.

#### RTP-preproc

The objective of the pre-processing is to correct the DWI measurements for known problems in the reconstruction. We implemented the pre-processing as a Docker Image and corresponding manifest. The algorithms themselves are adapted from MRtrix’s (https://github.com/MRtrix3/MRtrix3) recommended preprocessing procedure, and these are also implemented as containers in Brainlife (https://brainlife.io/app/5a813e52dc4031003b8b36f9). Code available at github.com/vistalab/rtp-preproc.

The parameters we used to perform the pre-processing are: (a) Diffusion data in 4D nifti format, (b) BVAL and BVEC (Bvec files in FSL format), and (c) Anatomical T1w files used to align the diffusion data. The Docker Image has additionally parameter settings that enable users with DWI data acquired using reverse phase-encoding methods to apply FSL’s TOPUP to correct for EPI geometric distortions. This option is particularly relevant for downstream analyses that require a precise alignment between anatomical and diffusion data (e.g., MRtrix’s Anatomically Constrained Tractography).

Next we provide a list of the preprocessing steps that can be set; the values used in the shared pipeline are between brackets (for WHL and YWM, for HCP check the references for the full parameter configuration):

1. rpe: specifies if there is reverse phase encoding data or not (false)
2. acqd: acquisition phase encoding direction (PA: Posterior > Anterior)
3. denoise: perform a principal component analysis (PCA) based denoising of the data^13,14^ (true)
4. degibbs: perform Gibbs ringing correction^15^ (true)
5. eddy: perform FSL’s eddy current correction; if inverted phase encoding direction files are found, eddy will be done as part of FSL’s topup^16^ (only eddy, as there is no reversed phase encoding data)
6. bias: compute bias correction with ANTs^17^ (true)
7. ricn: perform Rician background noise removal^18^ with custom code using MRtrix tools, please check repository for details (true)
8. norm: perform intensity normalization (false)
9. nval: normalise the intensity of the b = 0 signal within the FA-derived white matter mask to this number (does not apply)
10. align: align dwi data with AC-PC anatomy (true)
11. doreslice: do reslicing to the amount set in reslice (false)
12. reslice: if doreslice is true, reslice the DWI data to an isotropic voxel of this value (does not apply)

It has been our experience that on some occasions the orientation of the diffusion data from the scanner can be flipped (right/left, up/down). We implemented a deep neural network solution to detect if there has been a flip in the sign of any of the directions in the bvec files and corrects them. This algorithm is also implemented in Python^19–22^ as a Docker container. The code is publicly available in https://github.com/vistalab/RTP-bvec-flip-detect.

Finally, we validated the pre-processing by visualizing the pre-processed data either directly in the web browser window, or after downloading the images locally. The HCP dataset was preprocessed by that consortium^16,23,24^ and only the RTP-pipeline container was applied.

#### RTP-pipeline

**Fig. 2:**
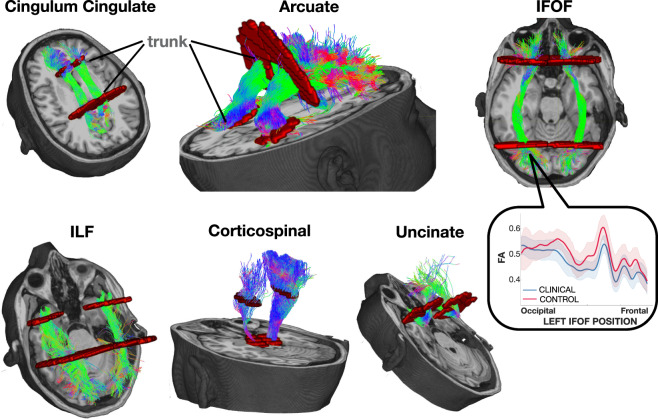
Six illustrative pairs of homologous tracts and their defining ROIs. The streamlines serve as a model of white matter tracts; they are selected by fitting to the diffusion weighted imaging (DWI) measurements. The tracts are defined by regions of interest (ROIs, red) that select specific streamlines from the whole brain tractograms. The region between the two ROIs is relatively stable and called the trunk. We estimate a core fiber from the collection of streamlines and sample 100 equally spaced segments. The FA of the core fiber is calculated by combining FA transverse to the core fiber at every sample point, using a Gaussian weighting scheme over distance. The set of sample points is the tract profile; the average of the FA values of the core fiber is the mean tract FA. In the inset, an illustrative example of two group profiles. The dark outline is the mean of the group and the shaded outline represents a standard deviation of the values.

The RTP-pipeline container takes preprocessed DWI data and automatically identifies 20 white matter tracts by default (see Fig. 2 for six illustrative tracts). The dataset we share includes eight different summary measures of the diffusion data measured at sample points along the length of the central path within each of these tracts (tract profiles). The inset in Fig. 2 is an example of two group mean FA tract profiles.

The diffusion modeling and tracking in RTP-pipeline is based on MRtrix, and the tract profiling and metric obtention software is based on Matlab code from mrVista and Automated Fiber Quantification^25^. For a complete list please refer to the manifest.json file in (github.com/vistalab/RTP-pipeline). The manifest.json contains a description and a default value per every parameter. It is important to notice that the parameter selection is project specific. Check github.com/vistalab/RTP-pipeline/wiki for more detailed information.

Every data row in the shared dataset includes a sub-table with the list of parameters used and the version of RTP-pipeline used to generate the values, so the provenance and the replicability of each data point is guaranteed. These are the main steps we followed in RTP-pipeline to generate the share data:

1. Brain and white matter mask creation.
2. Response function creation. We used the dhollander algorithm with automatic lmax calculation^26^. It separates the WM, GM, and CSF in the DWI without requiring an anatomical file.
3. Constrained Spherical Deconvolution (CSD) modelling. Different algorithms can be selected. It is performed at the voxel level and it can model crossing fibers. It creates Fiber Orientation Distributions (FOD) that are then used for tracking.
4. Whole brain white matter streamlines estimation. We used MRtrix’s tckgen function with the iFOD2 algorithm, combined with Ensemble Tractography^27^ and LiFE (Linear Fascicle Evaluation)^28^. The shared datatable can be checked for the specific parameters used.

- Ensemble Tractography (ET) method: ET invokes MRtrix’s tractography tool a number of times, constructing whole brain tractograms with a range of tracking parameter options. For example, the minimum angle parameters for tracking can be varied (e.g. 47, 23, 11, 6, 3), and the resulting whole brain connectomes concatenated together. There are config parameters specific to the Ensemble Tractography implementation.
- The LiFE (Linear Fascicle Evaluation) method evaluates the tractogram streamlines and retains those that meaningfully contribute to predicting variance in the DWI data. There are config parameters specific to the LiFE implementation.

5. We used a custom version of the AFQ method^25^, which segments streamlines into tracts (Fig. 2; the specific code can be checked in https://github.com/vistalab/RTP-pipeline/tree/3.0.6). The tracts are defined by regions of interest (ROIs, in red in Fig. 2) that select specific streamlines from the whole brain tractogram. Several options that control this step can be configured with the configuration parameters (see the data table for the parameters used).
6. DTI modeling at the voxel level, for DTI metric creation, such as FA, MD, RD…
7. Tract Profiles: the metrics are calculated for each individual tract creating the tract profiles. Although the most common ones are the DTI metrics (FA, MD, …), we have successfully used others, such as T1 relaxation time or macromolecular tissue volume (MTV) quantitative MRI maps, or the FOD values. In this dataset, we shared the most common ones based on the DTI model, and other related to the tract geometry (see Data Records for more detail). We used the following steps to extract the metrics from every fiber: (a) We identified a core fiber, representing the central tendency of all the streamlines in the tract; (b) We sampled equally spaced positions along the fiber between the two defining ROIs (N = 100); (c) We measured and combined the metric values of streamlines at locations transverse to each sample position. The combination is weighted by a Gaussian value based on the distance from the sample point. (d) The sampling and transverse averaging generates a tract profile of 100 metric values (see inset in Fig. 2 for a group mean FA tract profile).

## Data Records

We share the data in a figshare collection^29^, that contains the same information in two equivalent formats: a single Matlab table file and a nested json file. Both are curated and organized, ready for further analysis. There are examples of reading and using the data in https://github.com/garikoitz/paper-scidata.

### Data structure

The data set contains 10120 rows and 15 columns. Due to the complexity of the metadata, it has been shared in a nested structure: some variables are at the first level of the table, while others are organized in sub-tables. The description of the columns is as follow:

- **Proj:** Project description. In this case, we combined three different projects, which correspond to three different acquisition sites.
- **Struct:** Name of the white matter tract. We are sharing 20 white matter tracts. Check the dataset for the names.
- **SubjID:** Unique identifier for the subject. There can be multiple data associated with a single subject.
- **TRT:** TRT stands for Test-Retest. One dataset was repeated, meaning that the same subject was scanned twice. Repeated acquisitions are very useful to check the reproducibility of an acquisition.
- **ad:** 100 value vector with axial (longitudinal) diffusivity values. See Fig. 2.
- **fa:** 100 value vector with fractional anisotropy values. See Fig. 2.
- **md:** 100 value vector with mean diffusivity values. See Fig. 2.
- **rd:** 100 value vector with radial diffusivity values. See Fig. 2.
- **torsion:** 100 value vector with tract core Frenet-Serret torsion values, calculated by taking a weighted average on each fiber. See Fig. 2.
- **curvature:** 100 value vector with tract core Frenet-Serret curvature values, calculated by taking a weighted average on each fiber. See Fig. 2.
- **volume:** 100 value vector with tract volume values. It was calculated based on the number of unique voxel coordinates at each node. See Fig. 2.
- **SubjectMD:** This sub-table contains the metadata associated with the subject. There are 20 values with the most important subject information (gender, age…), as well as unique identifiers to locate the subject in the database. The info table within SubjectMD contains another 39 values with the subject’s behavioral information. This information is project dependent, as not all subjects have the same behavioral information.
- **AcquMD:** This sub-table contains the metadata associated with the MR acquisition: sequence, vendor, software version, etc. with 47 metadata values. There is one variable in AcquMD which is especially important for this dataset, scanbValue, that is the b value of the acquisition. We used it to separate datasets in our FA analysis in^1^. Many other variables (md, rd, gradient strength, number of directions…) have not been explored yet.
- **AnalysisMD:** This sub-table contains the metadata associated with the Analysis. Software used, version of the software, and all the config parameters used to obtain the data points. This sub-table contains information of 61 configuration parameters. These values are the same across all subjects. This parameter list, along with the container version used, guarantees that the same analyses can be performed in the same (for reproducibility), or new (for generalization) datasets. It is worth exploring whether the analysis parameters should be chosen based on the acquisition parameters.

The repository https://github.com/garikoitz/paper-reproducibility includes the analyses performed in^1^, which may server as a convenient starting point for further analyses.

### Data storage and provenance

The data stored in figshare were derived from DICOM files stored in the data and computation management system. These protected health information records are available to investigators with IRB approval. The data were extracted from the system and uploaded to figshare using the methods in this repository https://github.com/garikoitz/paper-scidata.

Using a programmatic method to extract the data guarantees that we can reproducibly retrieve the data stored in figshare. Every line of information in the database we are sharing contains unique identifiers to the data-objects in the Flywheel database. The provenance of the dataset is traceable from the original DICOM files to the figshare files.

## Technical Validation

Human *in-vivo* white-matter diffusion measurements require several steps of quality assurance and calibration. Tests are performed in the MRI scanners before the acquisitions. After the acquisition, quantitative and qualitative quality control is performed in the MRI DICOM images. Next, the images are preprocessed with RTP-preproc to correct for known and not desired distortions in the image, after which a second quality control is performed. Next, the images go to the RTP-pipeline where the white matter tracts are identified. The tracts in each individual subject are visualized in both 2D and 3D and inspected for major errors. To allow the between subject comparison, the parameter selection is made at the group level, but the tracts are checked at the individual level. After the tracts are checked, we obtain summary metrics, create tract profiles and add the metadata.

The retest data are an additional tool for technical validation. We can expect to find very similar results between the test and retest measurements. The results in^1^ can be considered as an additional validation check, as we perform said test-retest comparisons. We compare the test-retest values with the values in the other projects too, to check if we obtain similar white matter profiles and the absolute values are inside a reasonable range. As far as we know, there is no ground-truth based software validation system for diffusion MRI^30^.

## Usage Notes

### Python/R/others

We created a Python Notebook example in https://github.com/garikoitz/paper-scidata, called readNestedJson.ipynb. We recommend extracting only some of the nested variables as first level variables in the datatable/dataframe. The researcher can explore the metadata to select the ones she is most interested in. For example, scanbValue should be one of the values in the first level (see Box 1).

### Matlab

Once the Matlab table file has been downloaded from figshare, loading the file shows all its functionalities as it was originally created in Matlab (see Box 1). There are many useful functions to analyze this dataset in https://github.com/garikoitz/paper-reproducibility.

Box 1. Python

>>>    import pandas as pd

>>>    import numpy as np

>>>    import matplotlib.pyplot as plt

>>>    dt = pd.read_json(filename)

>>>    dt[:3] # See first 3 rows

>>>    # Extract the variable AcquMD.scanbValue to the first

>>>    # level to make it more useful

>>>    dt[‘bValue’] = pd.Series([dt[‘AcquMD’][i][0][‘scanbValue’] for i in dt.index], index = dt.index)

>>>    # Extract the fractional anisotropy (FA) values of

>>>    # Subject:036, Proj:YWM, Struct:LeftCorticospinal, b value:1000.

>>>    myFA1000 = dt.fa[(dt.bValue = = 1000) & (dt.Proj = = “YWM”) & (dt.SubjID =  = “036_HM”) & (dt. Struct = = “LeftCorticospinal”)]

>>>    # PLOT IT

>>>    x = np.arange(start = 1, stop = 101, step = 1)

>>>    fig = plt.figure()

>>>    ax = plt.axes()

>>>    plt.plot(x, myFA1000.to_numpy()[0], ‘-r’, label = ‘b = 1000’)

>>>    plt.show()

Matlab

>>    load(filename);

>>    head(DT)

>>    % Extract the fractional anisotropy (FA) values of

>>    % Subject:036, Proj:YWM, Struct:LeftCorticospinal, b value:1000.

>>    myFA1000 = DT{DT.SubjID = = “036_HM” & DT.Proj = = “YWM” & DT.AcquMD.scanbValue = = 1000 & DT.Struct = = ”LeftCorticospinal”,”fa”}

>>    plot(myFA1000)

**Docker and Singularity**.

To install the containers used in this analysis in Docker, use this in command line:

#$ docker pull garikoitz/rtp-pipeline:3.0.6

and for the Singularity installation use:

#$ singularity build rtp-pipeline_3.0.6.sif docker://garikoitz/rtp-pipeline:3.0.6

### How to cite

We recommend the users describe the dataset including at least the following information:

*“Diffusion metrics were obtained from 231 MRI sessions, 187 unique subjects, from three different sites. All data provided was obtained following the IRB guidelines of the relevant sites. The metrics of the white matter tracts were obtained using the Reproducible Tract Profiles algorithm. For further details see [this one, Lerma-Usabiaga (under review) Scientific Data]”*.

### How to extend this dataset

This dataset has been created with reproducible code that extracts information from the RTP tools and creates tables with the data and metadata. The main code used to extract the shared dataset can be found here: https://github.com/garikoitz/paper-scidata/blob/master/mainScript.m. To extend the data shared in this publication with another dataset run with RTP, they can use the script to create a table and just row-bind it to this one. Examples of new datasets: a new set of subjects run with the same RTP version, same or a portion of the dataset shared here but analyzed with different options, or the same dataset shared here run with the same options but with a different version of RTP (for test-retest analyses of the tools). The data is formatted in a way that it will be ready for analysis once it has been binded.

## Data Availability

All the code used in the generation of this dataset is in the public domain, and it has been linked in the corresponding sub-section in this manuscript. Here we provide a summary of the linked repositories: Main repository for this publication: https://github.com/garikoitz/paper-scidata. Useful examples on data handling: https://github.com/garikoitz/paper-reproducibility. Code to create the preprocessing container: https://github.com/vistalab/RTP-preproc. Code to create the tracking container: https://github.com/vistalab/RTP-pipeline. Code to systematically access data and create tables: https://github.com/vistalab/scitran/ The Docker containers with the algorithms and the configuration parameters can be run in a computer with Linux, macos (tested) or Windows (not tested). The Docker containers can be downloaded and run both in Docker and Singularity for free.
